# Eye-blink artifact removal from single channel EEG with *k*-means and SSA

**DOI:** 10.1038/s41598-021-90437-7

**Published:** 2021-05-26

**Authors:** Ajay Kumar Maddirala, Kalyana C Veluvolu

**Affiliations:** 1 School of Electronics Engineering, Kyungpook National University, Daegu, 41566 South Korea; 2 School of Electronic and Electrical Engineering, Kyungpook National University, Daegu, 41566 South Korea

**Keywords:** Computational neuroscience, Biomedical engineering, Electrical and electronic engineering

## Abstract

In recent years, the usage of portable electroencephalogram (EEG) devices are becoming popular for both clinical and non-clinical applications. In order to provide more comfort to the subject and measure the EEG signals for several hours, these devices usually consists of fewer EEG channels or even with a single EEG channel. However, electrooculogram (EOG) signal, also known as eye-blink artifact, produced by involuntary movement of eyelids, always contaminate the EEG signals. Very few techniques are available to remove these artifacts from single channel EEG and most of these techniques modify the uncontaminated regions of the EEG signal. In this paper, we developed a new framework that combines unsupervised machine learning algorithm (*k*-means) and singular spectrum analysis (SSA) technique to remove eye blink artifact without modifying actual EEG signal. The novelty of the work lies in the extraction of the eye-blink artifact based on the time-domain features of the EEG signal and the unsupervised machine learning algorithm. The extracted eye-blink artifact is further processed by the SSA method and finally subtracted from the contaminated single channel EEG signal to obtain the corrected EEG signal. Results with synthetic and real EEG signals demonstrate the superiority of the proposed method over the existing methods. Moreover, the frequency based measures [the power spectrum ratio ($\Gamma$) and the mean absolute error (MAE)] also show that the proposed method does not modify the uncontaminated regions of the EEG signal while removing the eye-blink artifact.

## Introduction

Electroencephalography (EEG) records brain electrical activity and it plays an important role in understanding of the motor functions, the cognitive loads, the level of attention and the brain disorders^1–5^. The EEG signals are widely used in application like brain computer interface (BCI) system, that extract the information from the EEG signals and send that as a command signal to a physical system. Several methods have been proposed to extract the EEG components for band identification in BCI application^6,7^. Recently, time varying complex network models are also proposed to enhance the classification accuracy of BCI systems^8,9^. In recent years, there is a great demand for in-home health monitoring due to increase in chronic illnesses. This demand brings a need for developing portable wireless healthcare systems to measure biomedical signals in home environment. With increasing advancement in technology, the healthcare systems were designed with low instrumentation complexity^10,11^. Recently, portable EEG devices with single EEG channel are widely used to measure the brain signals in non laboratory/clinical applications^12,13^. The use of these devices and their performance is also studied in different applications such as BCI, driver fatigue detection and brain disorders^14–18^.

In general, the EEG signals are often contaminated by several artifacts such as the electrooculogram (EOG), the electromyogram (EMG), electrocardiogram (ECG) and motion artifacts are a result of for example, EOG artifact is a result of an eye-blink activity. However, due to the involuntary movement of eyelids, the EOG artifact (from here onward we will be calling it as eye-blink artifact) always present in EEG signal. This eye-blink artifact appears as a high amplitude spike like signal in the EEG and contaminates it in time and frequency domains. More specifically, the eye-blink artifact contaminates the low-frequency EEG bands (0–12 Hz)^19^ which are associated to hand movements, attention levels and drowsiness^2,20,21^. However, inaccurate filtering of artifacts may effect the signal in both time and frequency domains and loss of information may result in compromising the end applications, for example, in the fatigue detection and BCI applications^14,15,17,18,22,23^.

Removal of eye-blink artifact has been a challenging task, as they often overlap with the lower frequency spectrum of EEG signal. The usage of traditional low-pass or band-pass filter for eye-blink artifact elimination may also remove some components of the actual EEG signal. The adaptive filters have been applied to remove eye-blink artifact from EEG signals^24^. However, they require a reference signal that some how correlates with the eye-blink artifact registered in the EEG signal. Similar to the principle component based method, an artifact subspace reconstruction (ASR) method was also proposed to remove artifacts from multichannel EEG data^25^. This method exploits the clean regions of the data as reference and removes the eye-blink components. Independent component analysis (ICA), is a blind source separation (BSS) technique used to decompose (separate) the sources from physiological signals^26,27^. However, ICA has been widely employed to remove artifacts from multichannel EEG signals^26,28,29^. In addition to this, canonical correlation analysis (CCA), another BSS based technique, is also popular to remove eye-blink artifacts from the multichannel EEG signals^30,31^. These two methods further integrated with other methods to improve their performance in removing artifacts^32–34^. The CCA method was successfully employed to remove muscle artifact from the EEG data^35,36^. The main difference between these two techniques lies in the extraction of source components from the mixed EEG data. The eye-blink artifacts are captured by most of the EEG channels as it is a high energy component and redundant information associated to the eye-blink artifact is present in the multi channel EEG data. The multichannel based artifact removal methods relies on this inherent advantage to extract the information and to remove the artifacts. Therefore, removing artifact from single channel EEG data is more challenging as compared to multi-channel EEG.

The ensemble empirical mode decomposition (EEMD)^37^ and ICA techniques are combined (also called EEMD-ICA), to separate the sources from single channel EEG signal using ICA^38^. In this method, the EEMD is employed to decompose single channel EEG signal into multi-variate data. After that, ICA algorithm is used to derive the demixing matrix and independent components (ICs) from the multivariate data. To extract the desired source signal, the corresponding column vector of mixing matrix (inverse of demixing matrix) is multiplied with that of IC of interest. Finally, the desired source signal can be obtained by summing all the components obtained in the multiplication step. Recently, singular spectrum analysis (SSA)^39^ has been successfully employed to decompose the EEG signals^40–42^. The SSA is jointly used with ICA to remove different artifacts from single channel EEG signals^43^. The main difference between EEMD-ICA and SSA-ICA techniques lies in the way they decompose the given single channel EEG signal into multivariate data. Surrogate based technique has been proposed to remove artifacts from single channel EEG signal^44^. However, this method can work only under the assumption of EEG stationarity, which may not hold for lengthy EEG epochs.

The physiology based technique to remove eye-blink artifact from single channel EEG signal was developed^45^. This method removes eye-blink artifact without altering the uncontaminated regions in the EEG signal. In this method, first, a skeleton of eye-blink artifact will be constructed using straight lines. Next, it will be shaped to the individual eye-blink detected based on the set threshold. However, the threshold will be determined based on the eye-blink potential of each individual. Moreover, this method is sensitive to high amplitude neural spikes and also fails to detect when the eye-blink artifact is mixed with other artifacts. Recently, the Fourier-Bessel series expansion based empirical wavelet transform (FBSE-EWT) is proposed to eliminate the eye-blink artifact from single channel EEG signal^46^. In this method, the EEG components, such as $\delta , \theta , \alpha$ and $\beta$ are extracted from single channel EEG signal using FBSE-EWT. The local polynomial approximation based total variation (LPATV) filtering is applied to the $\delta$ component. However, this method requires the optimization of several parameters such as regularization parameter, block length, number of overlapping, etc., for the accurate removal of eye-blink artifact.

Most of the artifact removal methods discussed above alters the EEG components in the non-artifact regions of the given EEG signal. To overcome the limitations of the existing methods, in this paper, we propose a new method, in which an unsupervised machine learning algorithm ($k$-means) and SSA technique are combined to remove eye-blink artifact from single channel EEG signal. In this method, first, the given single channel EEG signal is mapped into multivariate data matrix using embedding step of SSA. Next, four time domain features (the energy, the hjorth mobility^47^, the kurtosis and the difference between the maximum and minimum of the column vector of the multivariate data matrix) were computed and the $k$-means algorithm is employed. However, the novelty of the present work lies in the estimation of the eye-blink artifact using the time-domain features of the given EEG signal and the unsupervised machine learning algorithm. Finally, the estimated eye-blink artifact is further processed by SSA technique and subtracted from the contaminated EEG signal to obtain the corrected EEG signal. Simulations on both synthetic and real EEG data demonstrate the superior performance of the proposed method in removing eye-blink artifact from single channel EEG signals as compared to existing methods.

## Results

### Constructing synthetic EEG and eye-blink signals

**Figure 1:**
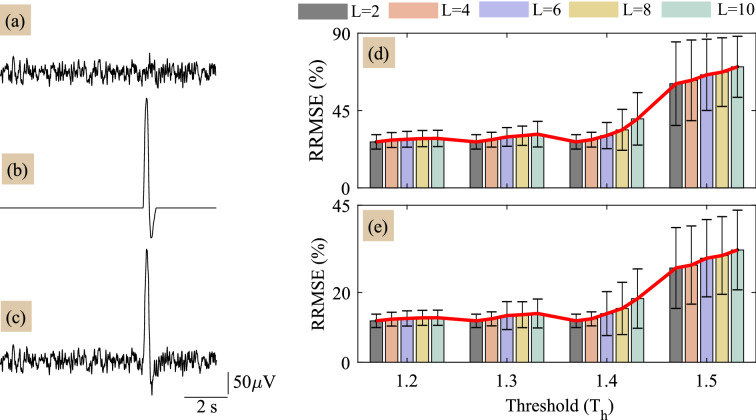
(**a**,**b**) ground truth EEG and eye blink artifact signals, respectively and (**c**) synthetically generated contaminated EEG signal with $p=1$. (**d**) and (**e**) illustrate the performance of proposed method in terms of RRMSE vs threshold for corrected EEG and the estimated eye-blink artifact, respectively.

To validate the performance of proposed method, we construct the single channel ground truth EEG signal and the eye-blink artifact as follows: The ground truth or true EEG signal is not available usually. Therefore, for simulation studies, the non-artifact EEG region from a lengthy EEG signal is considered as a ground truth EEG signal. The non-artifact EEG region in a lengthy EEG record, where no eye-blink artifact is present at least for 10 s duration, is manually identified and segmented. With the same procedure, 20 such artifact-free EEG epochs were constructed from six subject’s lengthy EEG records. Figure 1a shows 10 s ground truth EEG signal. To construct the ground truth eye-blink artifact and maintain the real morphology of eye-blink artifact we followed the procedure similar to^43^; first, manually we identified eye-blink artifact region in the real EEG signal and segmented. Next, zeros are padded to the eye-blink segment such that the signal length is equal to 10 s. Thereafter, the *MATLAB* *smooth* command is used to remove the EEG remnants resided on the eye-blinks and also to smooth the discontinuity in between the edges of eye-blink segment and the zero line. Three similar eye-blink artifacts from three subjects EEG are constructed. Synthetically constructed eye-blink artifact is shown in Fig. 1b. With 20 EEG and three eye-blink epochs, we have constructed sixty $(20 \times 3=60)$ synthetically contaminated EEG signals based on the mixing model given in (7) and is shown in Fig. 1c.

### Results with synthetic EEG data

The performance of any artifact removal method depends on its parameters. The proposed method has three parameters: window length *M*, threshold $T_{h}$ and the number of clusters *L*. In general, the eye-blink time period varies between $100{-}400\; \text{ms}$. In order to capture the on-set and the off-set events of the eye-blink artifact, we set the window length *M* to $500\; \text{ms}=0.5\times 256=128$ samples. We analyze the performance of proposed method for four arbitrary thresholds ($T_{h}$) and for various number of clusters $L=2, 4, 6, 8$ and 10. This analysis helps to identify the optimal choice of the parameters that results in good performance. Figure 1d,e shows the performance of proposed method for various selections of threshold ($T_{h}$) and clusters (*L*). After extensive simulation analysis, we identified low RRMSE values for the threshold selection in the range of 1.2 to 1.4 and clusters in the range of 2 to 4. However, we also observed sudden increase in the RRMSE for $T_{h}>1.4$. It is noticed from the simulation analysis that the threshold ($T_{h}$) acts similar to a cut-off frequency as in classic filters and whereas the number of clusters *L* will act as the number of decomposition levels as in a wavelet decomposition. Therefore, for better performance, we set the number clusters as *L* to 4 and the threshold $T_{h}$ to 1.4.

**Figure 2:**
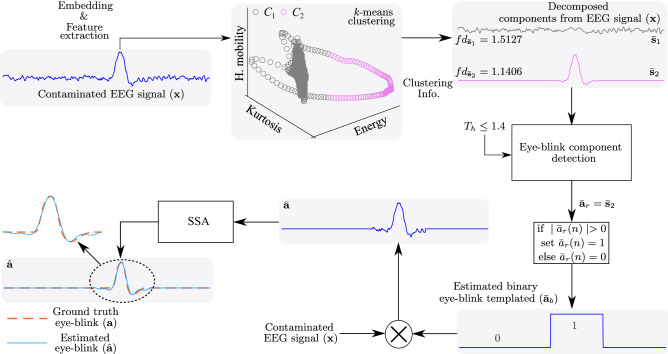
The key steps for estimating the eye-blink artifact $\hat{\mathbf {a}}$ from the contaminated EEG signal $\mathbf {x}$. Note that $fd_{\bar{\mathbf {s}}_{i}}$ represents the fractal dimension of $i$th signal $\bar{\mathbf {s}}_{i}$. Here, we have considered only three features for illustration.

Figure 2 shows the key steps to estimate the eye-blink artifact $\hat{\mathbf {a}}$ from the contaminated EEG signal $\mathbf {x}$. In order to estimate the eye-blink artifact $\hat{\mathbf {a}}$, first, the contaminated EEG signal $\mathbf {x}$ is embedded into a matrix $\mathbf {X}$ using (1), which results in *K* signal vectors of length *M* samples. Next, the four time domain features the energy, the hjorth mobility^47^, the kurtosis and the difference between the maximum and minimum of each column of $\mathbf {X}$ are computed. After performing the embedding and the feature extraction steps, the $k$-means clustering algorithm is applied. As the eye-blink artifact appears as a high amplitude and slow varying component in the contaminated EEG signal, the signal vectors corresponding to the eye-blink and EEG are well separated in the feature space as evident from Fig. 2. The $k$-means clustering algorithm assigns labels to each feature point in the feature space. This labeled information allows us to identify the cluster to which particular feature point (indirectly the column vector of $\mathbf {X}$) falls. Using (2), the matrices $\bar{\mathbf {X}}_{1}$ & $\bar{\mathbf {X}}_{2}$ are derived (here the number of clusters *L* is set to 2).

Next, uni-variate signals $\bar{\mathbf {s}}_{1}$ & $\bar{\mathbf {s}}_{2}$ are constructed using (6). The resulting decomposed uni-variate signals $\bar{\mathbf {s}}_{1}$ & $\bar{\mathbf {s}}_{2}$ are shown in Fig. 2. The fractal dimension of signals $\bar{\mathbf {s}}_{1}$ & $\bar{\mathbf {s}}_{2}$ are computed to identify the eye-blink artifact. After identifying the eye-blink artifact with pre-set threshold $T_{h}$, binary eye-blink template is constructed using step 6 of the proposed method (see “Methods” section) and multiplied with the contaminated EEG signal $\mathbf {x}$. With this, the eye-blink component $\bar{\mathbf {a}}$ mixed in the contaminated EEG signal $\mathbf {x}$ is extracted. However, the direct subtraction of this component from $\mathbf {x}$ results in zero line at the eye-blink region in the corrected EEG signal (which also means that the EEG components superimposed on the eye-blink artifact are also removed together with the artifact). Therefore, to smoothen the onset and offset regions of eye-blink artifact $\bar{\mathbf {a}}$, and to remove the EEG components superimposed on the eye-blink artifact, we employed SSA technique in this paper. We can see a smooth eye-blink artifact in Fig. 2 (zoomed region) that matches with the ground truth eye-blink artifact as indicated with black dotted lines. Finally, the estimated eye-blink artifact $\hat{\mathbf {a}}$ will be subtracted from the contaminated EEG signal to obtain the corrected EEG signal $\hat{\mathbf {s}}$.

**Figure 3:**
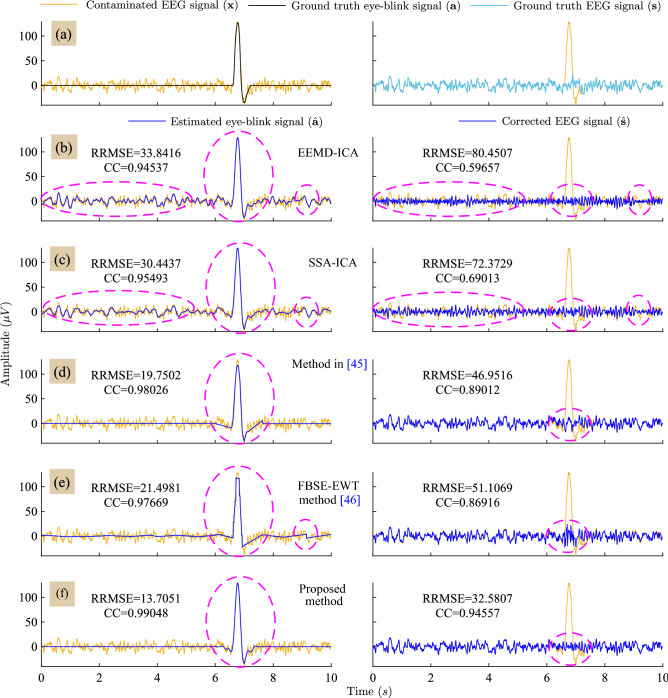
Row-wise: (**a**) Superposition of the ground-truth eye-blink artifact and the EEG signal ($\mathbf {s}$) with the contaminated EEG signal. (**b**)–(**f**) superposition of the obtained eye-blink artifact (left) and the corrected EEG signals (right) by all methods on the contaminated EEG signal (for $p=1$ case).

Figure 3 depicts the estimated eye-blink artifact ($\hat{\mathbf {a}}$) and the corrected EEG signal ($\hat{\mathbf {s}}$) obtained by all the methods. The RRMSE and CC values presented in Fig. 3b–f are computed with respect to the ground truth eye-blink ($\mathbf {a}$) and the EEG signal ($\mathbf {s}$) as shown in Fig. 3a. The proposed method has low RRMSE and high CC values as compared to the existing methods. However, we noticed abrupt changes at onset and offset regions of the eye-blink period and non-eyeblink periods in the estimated eye-blink artifacts obtained by the method in^45^ and FBSE-EWT methods, as evident from Fig. 3d,e in the first column. Moreover, the partial estimation of eye-blink artifact obtained by the methods in^45^ and the FBSE-EWT method can also be noticed in the same. As a result, eye-blink component can still be visualized in the corrected EEG signals obtained by the method in^45^ and the FBSE-EWT methods. In contrast, accurate estimation of the eye-blink artifact can be obtained with the proposed method and no artifact component can seen in the corrected EEG signal as shown in Fig. 3f. Even though the EEMD-ICA and SSA-ICA methods estimate the eye-blink component (eye-blink region between $6{-}8\;\text{s}$) accurately, however they alter the non-artifact regions, which is not desirable.

**Figure 4:**
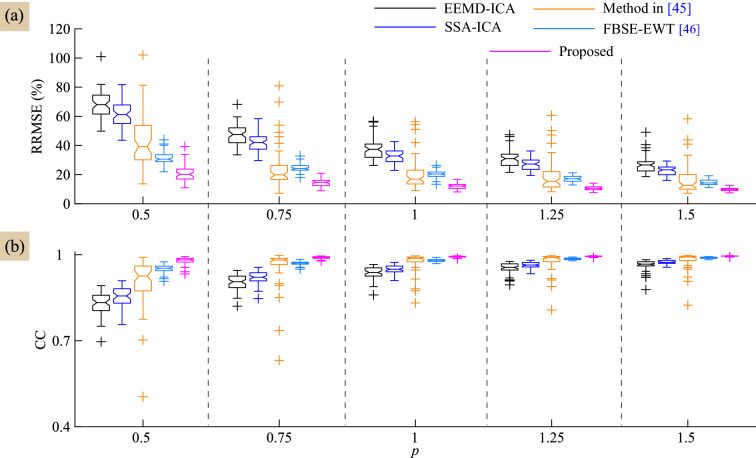
Performance comparison of all methods in terms of (**a**) RRMSE and (**b**) CC values with respect to the artifact mixing constant *p*.

As the SNR of the contaminated EEG signal varies with the artifact mixing constant *p*, we have evaluated the performance of the proposed and the existing methods for different *p* values. The variations of RRMSE and CC values of all methods with respect to *p* are shown in Fig. 4a,b. It is obvious from these results that the proposed method shows lower RRMSE and higher CC as compared to the existing methods. However, the mean RRMSE and CC values of the performance of proposed method are better compared with the existing methods, as evident from Table 1.

**Table 1:** Mean RRMSE and CC values of all method for various selections of artifact mixing constant *p*.

Method	$p=0.5$		$p=0.75$		$p=1$		$p=1.25$		$p=1.5$	
	RRMSE	CC	RRMSE	CC	RRMSE	CC	RRMSE	CC	RRMSE	CC
EEMD-ICA	67.8152	0.8297	47.3292	0.9041	37.4845	0.9358	31.2122	0.9529	26.8632	0.9642
SSA-ICA	61.1533	0.8522	41.8371	0.9219	32.5847	0.9499	27.2104	0.9640	22.7803	0.9746
Method in^45^	42.5856	0.9059	24.3101	0.9627	20.0368	0.9764	18.7325	0.9784	16.2730	0.9829
FBSE-EWT^46^	31.3118	0.9513	24.6072	0.9703	20.2033	0.9801	17.0667	0.9858	14.7092	0.9895
Proposed	20.7450	0.9795	14.4100	0.9902	11.8837	0.9933	10.5518	0.9947	9.7430	0.9954

**Figure 5:**
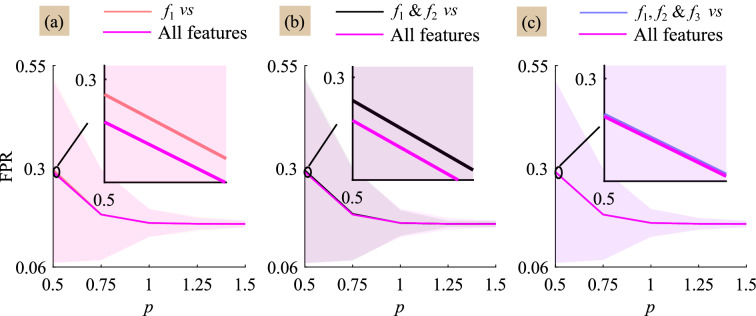
Comparison of averaged FPR curve obtained with four features vs (**a**) the energy feature (${f}_{1}$), (**b**) ${f}_{1}$ & the hjorth mobility (${f}_{2}$) and (**c**) ${f}_{1}, {f}_{2}$ & the kurtosis (${f}_{3}$) for each artifact constant *p*. The fourth feature is the difference of the maximum and minimum values (${f}_{4}$) of signal vector $\mathbf {x}^{i}, i=1, 2, \ldots , K$.

The performance of proposed method in detecting the eye-blink artifact is also studied by varying the feature selection. Figure 5 shows the detection of the eye-blink artifact with respect to the artifact mixing constant *p*. The detection of eye-blink artifact using the proposed method is evaluated in terms of False positive rate (FPR) and it is expected to be as small as possible. When the amplitude of eye-blink artifact is large i.e.,  $p\ge 1$, we do not see much improvement in FPR values with four features as compared to $f_{1}$, $f_{1}$ & $f_{2}$ and $f_{1}, f_{2}$, & $f_{3}$ feature selection. However, it is observed from Fig. 5a,b that the use of four time domain features helps in the improvement of the eye-blink artifact detection rate as compared with single and two time domain features for $p=0.5$. We do not seen much difference in FPR values obtained with the number of features 3 and 4 (Fig. 5c). Finally, from this study we observed that the energy features $f_{1}$ and $f_{4}$ contributes more towards detection of the eye-blink artifact as compared to other feature components. In Fig. 5, the FPR comparison plots for all features vs $f_{2}$, and $f_{3}$ have not showed, as the contribution of these components in detecting the eye-blink artifact is minimal.

We have also evaluated the performance with two different clustering algorithms—spectral clustering and agglomerative clustering. Spectral clustering algorithm does not show much improvement whereas an improvement can be seen with agglomerative clustering algorithm as compared to $k$-mean clustering algorithm. However, the agglomerative clustering algorithm’s computational complexity is high and it increases with the number of feature samples as compared to $k$-means clustering method.

### Results with real EEG data

**Figure 6:**
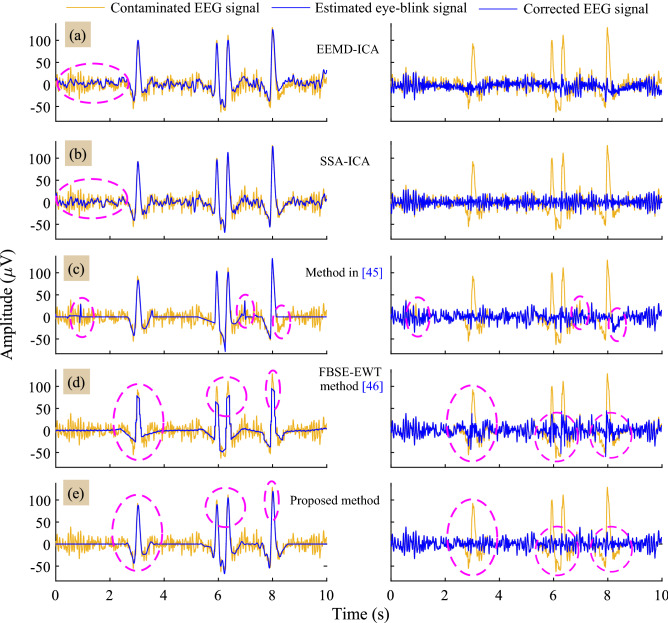
Row-wise: (**a**)–(**e**) superposition of the estimated eye-blink artifact (left) and the corrected EEG signals (right) of all the methods with the contaminated EEG signal.

For analysis with real EEG data, sixty 10*s* EEG epochs were segmented from the lengthy EEG data of twelve subjects such that at least one eye-blink is present in the EEG signal. Figure 6 shows the estimated eye-blink artifact ($\hat{\mathbf {a}}$) and the corrected EEG signal ($\hat{\mathbf {s}}$) by all methods. When EEMD-ICA and SSA-ICA are applied to the real EEG data, the low frequency EEG information is also extracted together with the eye-blink artifact (non-artifact regions in Fig. 6a,b first column). Few spikes and the partial extraction of eye-blink component at time period 8 s can be seen in the estimated eye-blink artifact by all the method^45^ (Fig. 6c first column). Similarly, we can see the clipping of the eye-blink component in estimated eye-blink artifact by the FBSE-EWT method (Fig. 6d first column). In contrast, spikes and partial removal of eye-blink component are absent with the proposed method (Fig. 6e first column). More importantly, the proposed method removes the eye-blink artifact without altering the EEG information from the non-artifact region of the EEG signal.

**Figure 7:**
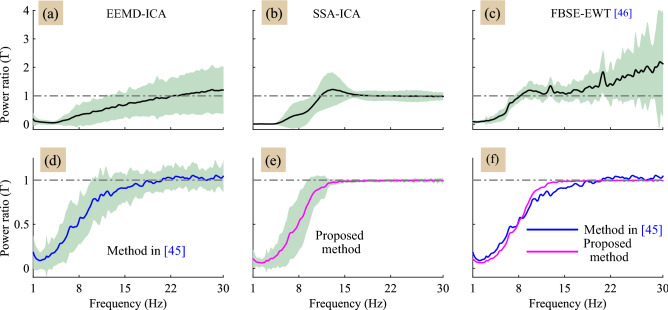
(**a**)–(**e**) shows the power spectral ratio curves of all the methods, and (**f**) the super position of the mean power spectral ratio curves of the proposed method and the existing method^45^.

In general, the ground truth EEG signal is not available to evaluate the performance of artifact removal methods on real EEG data. Therefore, we consider two metrics, the power spectral ratio, $\Gamma (f)$ and the MAE, to analyze the performance on real EEG signals. Figure 7, shows the average power spectral ratio curves for 60 EEG signals. The shaded region in Fig. 7a–e shows the standard deviation error from mean. It is clear from the power spectral ratio plots shown in Figure 7a–d that the existing methods alters the $\beta$ band components of the EEG signal (i.e. $12{-}30\;\text{Hz}$). Compared with the other methods, the method in^45^ shows comparable performance with the proposed method. Therefore, for comparative analysis, the mean power spectral ratio curves for proposed and method^45^ are shown in Fig. 7f. We have also computed the MAE for different bands to see the affect the artifact removal method in each EEG band. However, average MAE values obtained by the proposed method for the $\beta$ band are more good as compared to existing methods, as evident from the power spectral ratio plots shown in Fig. 7 and the Table 2.

**Table 2:** Mean MAE values of the proposed and the existing methods in different EEG bands $1{-}8,\;8{-}12$ and $12{-}30 \; \text{Hz}$.

Method	MAE		
	1–8 Hz	8–12 Hz	12–30 Hz
EEMD-ICA	19.4213	1.3561	0.1399
SSA-ICA	20.9366	1.1344	0.0815
Method in^45^	18.9892	0.7360	0.0457
FBSE-EWT^46^	18.5890	0.3428	0.0955
Proposed	19.6776	0.7495	0.0093

## Discussion

The performance of the proposed method in comparison with other existing methods is analyzed in terms of RRMSE and the CC values computed with respect to the ground truth eye-blink artifact (are shown in Fig. 4a,b). In all conditions i.e. $p=0.5, 0.75, 1, 1.25$ and 1.5, the mean RRMSE and CC values of the proposed method are different from the RRMSE and CC valuable of EEMD-ICA, SSA-ICA and the method^45^. Comparing the estimated eye-blink artifacts from synthetic and real EEG signals, the existing method^45^ fails to extract the initial and final change-over points (inflections) of the eye-blink artifact. The probable reason for this is that, the eye-blink artifact is constructed by the straight lines and the intersection of these lines are fitted with zero slope to EEG trace. Therefore, partial separation in change-over points of eye-blink can be seen in the corrected EEG signal (please refer to second column in Figs. 3d and 6c). Although there is no significant difference in the CC values obtained for the proposed and the FBSE-EWT methods for the conditions $p>1$, it can be clearly seen that the peaks of eye-blink component were clipped-off in the estimated eye-blink artifact obtained by the FBSE-EWT method (see the first column in Figs. 3e and 6d). As a result, we can see abrupt changes in the corrected EEG signal obtained by the FBSE-EWT method (see the second column in Figs. 3e and 6d). These changes will lead to modification in the spectrum of the corrected EEG signal.

In order to show the affect of artifact removal methods in the spectral domain, we computed the power spectrum ratio and the MAE. From the power spectral ratio plots it can be seen that all the methods removed the eye-blink artifact efficiently. However, from the power spectrum ratio plots we can notice that most of the existing methods alters the $\beta$ band ($12{-}30 \; \text{Hz}$) of the corrected EEG signal (Fig. 7a–d). In contrast, the proposed method does not alter the original EEG components and as a result the power spectrum ratio value is 1 in this frequency band (Fig. 7e). Hence, the MAE values obtained by proposed method in the $\beta$ band are more better as compared to the MAE values obtained by other methods.

From this study we observe that most of the existing methods alters the EEG components from the non-artifact regions of the EEG signal and as a result the spectrum of the corrected EEG signal is altered. In contrast to the existing methods, the proposed method exploited the difference in the time-domain features of the EEG signal (evident from Fig. 2) to remove the eye-blink artifact without altering the EEG components. Moreover, we also studied the eye-blink artifact detection rate in terms of FPR and found that the energy based features contributed more towards detection of the eye-blink artifact as compared to other feature components. As evident from the simulation studies, the performance of the proposed method is sensitive to the threshold $T_{h}$ selection. A careful selection is required and this is also the limitation of the proposed method. A statistical based approach to address the limitation of proposed method with respect to the threshold selection will be the topic of our future research. In this paper, we mainly focused on removing the eye-blink artifact using a simple unsupervised clustering algorithm. Hence, in this study, we have used $k$-means clustering algorithm over other methods as it is simple and computationally efficient unsupervised clustering algorithm. However, based on the requirement of the application, other clustering algorithms can also be employed in the proposed method.

## Conclusion

A new method that relies on the EEG time-domain features and the strength of unsupervised machine learning algorithm ($k$-means) followed by SSA technique was developed for accurate removal of the eye-blink artifact for the single channel EEG signal. Simple four time-domain features of the EEG signal were employed to estimate eye-blink artifact and SSA technique was employed to remove the EEG remnants on the estimated eye-blink artifact. Results show that proposed method removes the eye-blink artifact without altering the original EEG components. As the time domain features are good in differentiating the EEG and eye-blink components, the proposed method was able to remove the artifact from the EEG signals by proper tuning of its parameter (threshold $T_{h}$). Comparative analysis with existing methods also demonstrate the superiority of the proposed method in accurate filtering of eye-blink artifacts. The developed technique can be employed for EEG pre-processing in applications where fewer or single frontal EEG channel(s) are usually employed.

## Methods

### Proposed method

**Figure 8:**
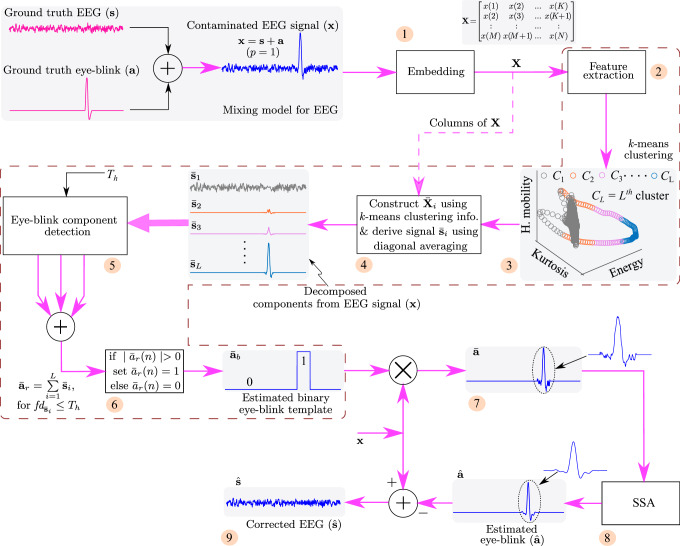
Block diagram of the proposed method (for $p=1$ case).

The overview of the single channel EEG artifact removal process employed in this paper is shown in Fig. 8. The novelty of the work lies in decomposing the given single channel EEG into *L* signals using the EEG features and the $k$-mean clustering algorithm. After decomposing the given single channel EEG signal into components, the fractal dimension (FD) of each decomposed component is computed. As the eye-blink artifact is a slow varying component, the FD is expected to be a small for the signals corresponding to the eye-blink artifact and is high for the signals corresponding to EEG. The eye-blink artifact component will be constructed by adding the decomposed components whose FD values are less than the pre-set threshold. Finally, the resulted eye-blink artifact component is further processed by SSA and subtracted from the contaminated EEG signal to obtain the corrected EEG signal. The proposed method relies on the following key steps to remove eye-blink artifact from single channel EEG signal.

#### Step-1, 2 and 3

Consider $\mathbf {x}=[x(1), x(2), \ldots , x(N)]=\mathbf {s}+p\mathbf {a}$ is an *N* sampled contaminated single channel EEG signal. The constant *p* represents the contribution of eye-blink artifact in the EEG signal, which we call it as artifact mixing constant. The signal vectors $\mathbf {s}$ and $\mathbf {a}$ represents the ground truth EEG signal and the eye-blink artifact, respectively. In this step, the given single channel EEG signal $\mathbf {x}$ is mapped into multivariate data matrix as in (1).

1 $$\begin{aligned} \mathbf {X}=\left[ \begin{array}{ccccc} x(1) &{} x(2) &{} ...... &{} ..... &{} x(K)\\ x(2) &{} x(3) &{} ..... &{} ..... &{} x(K+1)\\ : &{} : &{} ..... &{} ..... &{} :\\ x(M) &{} x(M+1) &{} ..... &{} ..... &{} x(N) \end{array}\right] =[\mathbf {x}^{1}, \mathbf {x}^{2}, \ldots , \mathbf {x}^{K}]=\mathbf {S}+\mathbf {A},  (p=1) \end{aligned}$$

where *M* is the window length and $K=N-M+1$. In $\mathbf {x}^{j}$ is the $j \text{th}, j=1, 2, \ldots , K$ column vector of $\mathbf {X}$. Note that in (1), we assumed the artifact mixing constant $p=1$ for simple understanding of the proposed method.

In step-2, four time domain features the energy (${f}_{1}$), the hjorth mobility^47^ (${f}_{2}$), the kurtosis (${f}_{3}$), and the difference in the maximum and minimum values of column of $\mathbf {x}_{i}$ (${f}_{4}=max\{\mathbf {x}^{i}\}-|min\{\mathbf {x}^{i}\}|$), are computed that results EEG feature data matrix $\mathbf {F}=[\mathbf {f}^{1}, \mathbf {f}^{2}, \ldots , \mathbf {f}^{K}]$ of size $4 \times K$, where $\mathbf {f}^{j}=[{f}_{1}^{j}, {f}_{2}^{j}, {f}_{3}^{j}, {f}_{4}^{j}]$ is $j$th column vector of feature matrix $\mathbf {F}$. Usually, the eye-blink artifact appears in the EEG signal as a high amplitude and slow varying component. The selected four features are good in extracting such inherent property of the eye-blink artifact.

In step-3, an unsupervised machine learning algorithm, i.e. $k$-means^48^ clustering algorithm, is applied on the extracted feature data matrix $\mathbf {F}$ with *L* number of clusters. The $k$-means clustering algorithm will provide the labels to each feature vector $\mathbf {f}^{j}$. The labeled information (clustering information) convey that to which cluster the feature vector $\mathbf {f}^{j}$ has fallen into.

#### Step-4

Based on the information obtained from the $k$-means clustering algorithm, first, multivariate data matrix $\bar{\mathbf {X}}_{i}, i = 1, 2, \ldots , L$ is constructed as follows. The $j$th column vector $\mathbf {x}^{j}$ of $\mathbf {X}$ is placed (copied) in the $j$th column of matrix $\bar{\mathbf {X}}_{i}$ when the $j$th feature vector $\mathbf {f}^{j}$ of matrix $\mathbf {X}$ belongs to $i$th cluster $C_{i}$; otherwise a vector with zeros is placed (copied) in that column of matrix $\bar{\mathbf {X}}_{i}$. This operation is shown with a dotted line from the output the embedding block in Fig. 8 and can be represented mathematically as

2 $$\begin{aligned} \bar{\mathbf {x}}_{i}^{j} = {\left\{ \begin{array}{ll} \mathbf {x}^{j} &{} \text{ if } \mathbf {f}^{j} \in C_{i},  \{i=1, 2, \ldots , L\} \\ \mathbf {0} &{} \text{ if } \mathbf {f}^{j} \notin C_{i}\end{array}\right. } \end{aligned}$$

where $\bar{\mathbf {x}}^{j}_{i}$ $(j=1, 2, \ldots , K)$, is the $j$th column vector of $\bar{\mathbf {X}}_{i}$.

Next, each $\bar{\mathbf {X}}_{i}$ matrix corresponding to each cluster is mapped into uni-variate signal using diagonal averaging step of SSA (using (6)). This results in *L* decomposed components, say $\bar{\mathbf {s}}_{1}, \bar{\mathbf {s}}_{2}, . . , \bar{\mathbf {s}}_{L}$ from the contaminated EEG signal $\mathbf {x}$. However, a criteria has to be selected to identify the signals corresponding to the eye-blink artifact from the *L* number of signals. A signal complexity measure, also called fractal dimension (FD)^49^, is successfully applied^50^ to identify the eye-blink artifact component from the estimated sources.

#### Step-5, 6 and 7

Hence, in step-5, we have computed FD for all *L* the decomposed components. Since the eye-blink artifact is characterized as high amplitude and slow varying component, the FD is expected to be a lower value for eye-blink artifact when compared to EEG signals. Finally, the decomposed components whose FD is less than or equal to the pre-set threshold ($T_{h}$) are added together. This results an eye-blink artifact component $\bar{\mathbf {a}}_{r}$. It is clear from (2) that some of the columns in $\bar{\mathbf {X}}_{i}$ are expected to be zeros. Applying diagonal averaging step of SSA on $\bar{\mathbf {X}}_{i}$, the amplitude levels in the reconstructed eye-blink signal are effected. In other words, there will be amplitude reduction in the eye-blink regions due to this diagonal averaging operation, as few of the columns of the matrix $\bar{\mathbf {X}}_{i}$ are zeros. Therefore, the direct subtraction of $\bar{\mathbf {a}}_{r}$ component from the contaminated EEG signal $\mathbf {x}$ will result the partial separation of eye-blink artifact.

To overcome such problem, in Step-6, each positive and negative valued samples in the obtained eye-blink artifact $\bar{\mathbf {a}}_{r}$ are replaced to one. This results in the formation of a binary artifact template $\bar{\mathbf {a}}_{b}$, whose sample values are ones in the artifact region and zeros in non-artifact region. In Step-7, the obtained binary eye-blink artifact template is multiplied with the input signal $\mathbf {x}$. Thus it results an eye-blink artifact component $\bar{\mathbf {a}}$ that is present in the contaminated EEG signal $\mathbf {x}$, with no amplitude changes in the artifact region.

#### Step-8 and 9

However, the obtained artifact component $\bar{\mathbf {a}}$ has some EEG remnants on the eye-blinks (Fig. 8). The direct subtraction of this component from the contaminated EEG signal results in the loss of valuable EEG information and a zero line can be seen in the corrected EEG signal. Therefore, the EEG remnants should be filtered (indirectly we are retaining the EEG components) before it is subtracted from the contaminated EEG signal $\mathbf {x}$.

In Step-8 of proposed method, we employ SSA (discussed in the following subsection) to remove the EEG remnants that reside on the eye-blink artifact component $\bar{\mathbf {a}}$. SSA can remove such remnants from eye-blink regions, as it relies on the co-variance of the data to separate the components. Therefore, the eye-blink artifact component $\bar{\mathbf {a}}$ is given as input signal to SSA and the EEG remnants are filtered out results an estimated eye-blink component $\hat{\mathbf {a}}$. Finally, in Step-9, the estimated eye-blink artifact signal $\hat{\mathbf {a}}$ will be subtracted from the contaminated EEG signal to obtain the corrected EEG signal $\hat{\mathbf {s}}$.

In the proposed method, the threshold $T_{h}$ plays an important role in identifying the eye-blink artifact component from the decomposed components after clustering. However, when there is no eye-blink artifact present in the EEG signal, it is obvious that all decomposed component belongs to EEG signal and the fractal dimension (FD) of these components is above the pre-set threshold $T_{h}$. When the FD of each component is above the threshold $T_{h}$, then step 6, 7,  and 8 of the proposed method are not performed and the estimated eye-blink artifact $\hat{\mathbf {a}}$ is set to zero. Finally, the corrected EEG $\hat{\mathbf {s}}=\mathbf {x}$, indicates that the given EEG signal is not contaminated by the eye-blink artifact.

### Singular spectrum analysis (SSA)

In this subsection, we briefly discuss about the SSA technique, as the proposed method development relies on the key steps of SSA technique. It is a data-driven subspace based technique, widely used to extract the low frequency trends and oscillating components from the noisy data^39,51^. Recently, SSA technique has been widely used to process several physiological signals^41,52,53^. Basically, it comprises the following steps: (i) embedding, (ii) decomposition, (iii) grouping and (iv) diagonal averaging.

#### Embedding

Consider $\bar{\mathbf {a}}=[\bar{a}(1), \bar{a}(2), \ldots , \bar{a}(N)]=\mathbf {a}+\mathbf {b}$, is a *N* sampled signal with noise. The vectors $\mathbf {a}$ and $\mathbf {b}$ represents the signal of interest and the noise component, respectively. In embedding step of SSA, the given uni-variate time-series signal $\bar{\mathbf {a}}$ is mapped into multi-variate data as shown below:

3 $$\begin{aligned} \bar{\mathbf {A}}=\left[ \begin{array}{ccccc} \bar{a}(1) &{} \bar{a}(2) &{} ...... &{} ..... &{} \bar{a}(K)\\ \bar{a}(2) &{} \bar{a}(3) &{} ..... &{} ..... &{} \bar{a}(K+1)\\ : &{} : &{} ..... &{} ..... &{} :\\ \bar{a}(M) &{} \bar{a}(M+1) &{} ..... &{} ..... &{} \bar{a}(N) \end{array}\right] =[\bar{\mathbf {a}}_{1}, \bar{\mathbf {a}}_{2}, \ldots , \bar{\mathbf {a}}_{K}]={\mathbf {A}}+\mathbf {B} \end{aligned}$$

where *M* is the window length and $K=N-M+1$.

#### Decomposition

In the next step, the singular value decomposition (SVD) of $\bar{\mathbf {A}}$ will be performed to decompose the trajectory matrix $\bar{\mathbf {A}}$ into $\bar{\mathbf {A}}_{1}, \bar{\mathbf {A}}_{1}, \ldots , \bar{\mathbf {A}}_{M}$. The *M* trajectory matrices can be obtained as follow: The SVD of a rectangular matrix of size $M \times K$ can be factored as $\mathbf {\bar{\mathbf {A}}}= \mathbf {U}\mathbf {D}\mathbf {V}^{T}$, where $\mathbf {U}$ and $\mathbf {V}$ represents orthogonal matrices, whose columns are eigenvectors of co-variance matrix $\mathbf {C}=\bar{\mathbf {A}}\bar{\mathbf {A}}^{T}$ and $\mathbf {C}=\bar{\mathbf {A}}^{T}\bar{\mathbf {A}}$, respectively; and the $\mathbf {D}$ is a rectangular diagonal matrix, whose elements are singular values ($\sigma$). Let the $\lambda _{1}, \lambda _{2}, \ldots , \lambda _{M}$ and $\mathbf {u}_{1}, \mathbf {u}_{2}, \ldots , \mathbf {u}_{M}$ represent the eigenvalues and the eigenvectors of the co-variance matrix $\mathbf {C}=\bar{\mathbf {A}}\bar{\mathbf {A}}^{T}$. Assume that the eigenvalues are sorted in the descending order based on their strengths (amplitudes), i.e. $\lambda _{1}\ge \lambda _{2}\ge , \ldots , \ge \lambda _{M}\ge 0$. Then, the $i$th trajectory matrix $\bar{\mathbf {A}}_{i}$ can be represented as

4 $$\begin{aligned} \bar{\mathbf {A}}_{i}=\sqrt{\lambda _{i}}\mathbf {u}_{i}\mathbf {v}_{i}^{T}  i=1, 2, \ldots , M \end{aligned}$$

where $\mathbf {v}_{i}=\bar{\mathbf {A}}^{T}\mathbf {u}_{i}/\sqrt{\lambda _{i}}$. Substituting $\mathbf {v}_{i}$ in (4), then the $i$th trajectory matrix $\bar{\mathbf {A}}_{i}$ is given by

5 $$\begin{aligned} \bar{\mathbf {A}}_{i}=\mathbf {u}_{i}\mathbf {u}_{i}^{T}\bar{\mathbf {A}} \end{aligned}$$

The term $\mathbf {u}_{i}\mathbf {u}_{i}^{T}$ in (5) form subspace for the $i$th component in the given signal $\bar{\mathbf {a}}$.

#### Grouping

The grouping step involves identifying the subspace for the desired signal (smooth eye-blink artifact in this present study). In other words, it identifies the appropriate eigenvectors (basis functions) to construct the desired signal. In the proposed method, we employ eigenvalue (spectrum) based grouping criteria to identify the desired eigenvector. Consider that the desired signal subspace can be constructed with *d* number of eigenvectors. To identify the *d* most significant eigenvectors (basis functions of desired signal), eigenvalue ratio is $r_{\lambda }(i)$ is computed by dividing each eigenvalue by the sum of total eigenvalues and is defined as $r_{\lambda }(i)=\lambda _{i}/\sum _{l=1}^{M}\lambda _{l}, i=1, 2, \ldots , M$. The indices of eigenvalues whose ratio greater than preset threshold $T_{SSA}$ (set to 0.01) are identified. The number of eigenvalues whose ratio is greater than $T_{SSA}$ is denoted as *d*. Finally, using (5) the *d* number of $\bar{\mathbf {A}}_{k}, k=1, 2, \ldots , d$ were computed and summed together. This results interested signal trajectory matrix in the form of $\hat{\mathbf {A}} =\sum _{k=1}^{d}\bar{\mathbf {A}}_{k}$.

#### Diagonal averaging

However, the obtained trajectory matrix $\hat{\mathbf {A}}$ do not hold the hankel structure to reconstruct uni-variate signal from it. Therefore, in the diagonal averaging (it’s a reverse process to the embedding step) step, the anti-diagonal elements of $\hat{\mathbf {A}}$ are replaced with their mean value as defined in (6). Lets consider that $\hat{a}(i,j)$ represents the $i$th row and $j$th column element of matrix $\hat{\mathbf {A}}$, then the desired signal can be estimated as follows

6 $$\begin{aligned} \hat{a}(n)= {\left\{ \begin{array}{ll} \frac{1}{n}\sum \limits _{i=1}^{n}{\hat{a}}(i,n-i+1)&{}\text {for}\, 1 \ge n< M\\ \frac{1}{M}\sum \limits _{i=1}^{M}{\hat{a}}(i,n-i+1)&{}\text {for}\, M \ge n \le K\\ \frac{1}{N-n+1}\sum \limits _{i=n-K+1}^{N-K+1}{\hat{a}}(i,n-i+1)&{}\text {for}\, K < n \le N \end{array}\right. } \end{aligned}$$

It is clear from (6) that the $n$th sample of estimated eye-blink artifact $\hat{a}(n)$ is an average of anti diagonal elements $\hat{a}(i,n-i+1)|_{i=1,2\ldots n}$, (for $n=2$, $\hat{a}(2)=\{\hat{a}(1,2)+\hat{a}(2,1)\}/2$). Finally, the corrected EEG signal $\hat{\mathbf {s}}$ is obtained by subtracting the estimated eye-blink artifact $\hat{\mathbf {a}}$ from the contaminated EEG signal $\mathbf {x}$.

### Performance measures

In this section, we have defined some measures to validate the performance of the proposed method over the existing methods. However, in this paper, we have considered the following EEG mixing model for analysis. Let the vectors $\mathbf {s}$ and $\mathbf {a}$ represent the ground truth EEG and the eye-blink artifact, respectively. Then the contaminated EEG signal $\mathbf {x}$ is defined as

7 $$\begin{aligned} \mathbf {x}=\mathbf {s}+p\mathbf {a} \end{aligned}$$

where *p* is constant that represents the contribution of eye-blink artifact in the contaminated EEG signal (known as artifact mixing constant). When $p>1$ (eye-blink artifact is more predominant) the signal to noise ratio (SNR) of contaminated EEG is low and when it is $<1$ SNR is high.

The performance of the proposed artifact removal method is evaluated on both synthetic and real EEG signals. In order to compare the performance of proposed artifact removal method with the existing methods on synthetic EEG signals, we have defined the following two performance measures: relative root mean square error (RRMSE) and correlation coefficient (CC). When the artifact removal method accurately estimates the eye blink artifact, the RRMSE and CC values between the ground truth and the estimated eye-blink artifacts are expected to be 0 and 1, respectively.

For simulation results with real EEG data, as neither ground truth EEG and nor the eye-blink artifact are usually available, we have defined two measures, power spectrum ratio $\Gamma (f)$ and mean absolute error (MAE) to evaluate the performance of all the methods. When the eye-blink artifact is perfectly removed from the contaminated EEG signal, $\Gamma (f)$ and the MAE are expected to be 1 and 0, respectively in the $\beta$ band ($12{-}30\; \text{Hz}$) of EEG signal. The power spectral ratio and MAE measures can objectively quantify if a particular method filters the artifact without altering the actual EEG signal.

#### Relative root mean square error (RRMSE)

Considering the ground truth or true eye-blink artifact as $\mathbf {a}$ and the estimated eye-blink artifact obtained by the artifact removal method as $\hat{\mathbf {a}}$, the RRMSE can be defined as

8 $$\begin{aligned} RRMSE=\sqrt{\frac{\sum \nolimits _{n=1}^N{[a(n)-\hat{a}(n)]}^{2}}{\sum \nolimits _{n=1}^Na^{2}(n)}} \times 100 (\%) \end{aligned}$$

where, *N* is the number of samples in the signal. When an artifact removal method accurately estimates the eye-blink artifact from the EEG signal, the difference between $\mathbf {a}$ and $\hat{\mathbf {a}}$ (numerator term) will be small and hence RRMSE value is expected to be small for a good artifact removal method.

#### Correlation coefficient (CC)

$\textit{CC}$ is a statistical based performance measure, represents the correlation between two signals. The CC between the ground truth eye-blink $\mathbf {a}$ and the estimated eye-blink $\hat{\mathbf {a}}$ components is defined as

9 $$\begin{aligned} \textit{CC}=\frac{cov(\mathbf {a},\hat{\mathbf {a}})}{\sigma _{\mathbf {aa}}\sigma _{\hat{\mathbf {a}}\hat{\mathbf {a}}}} \end{aligned}$$

The correlation coefficient between the ground truth and the estimated eye-blink artifact is expected to be 1 when an artifact removal method perfectly estimated the eye-blink artifact.

#### Power spectrum ratio ($\Gamma$)

It is a plot describing the ratio of the power spectrum of the corrected EEG signal to the power spectrum of the contaminated EEG signal and is used as metric to evaluate the performance of the proposed technique on real EEG signals^54^. The power spectrum ratio of the corrected and the contaminated EEG signals at each frequency is defined as

10 $$\begin{aligned} \Gamma (f)=\frac{p_{\hat{\mathbf {s}}}(f)}{p_{\mathbf {x}}(f)}, \quad f=1, 2, \ldots , 30, \end{aligned}$$

where, $p_{\hat{\mathbf {s}}}$ and $p_{\mathbf {x}}$ are represents the power spectrums of the corrected and the contaminated EEG signals, respectively. In general, the energy of eye-blink artifact lies in the band between 0 and 12 Hz. However, when the eye-blink artifact is removed, the power spectrum ratio of corrected EEG signal to the contaminated EEG signal is expected to be less than 1 in the frequency band 0–12 Hz. The low value of $\Gamma (f)$ in this band doesn’t mean the EEG components in 0–12 Hz band are removed, rather it is due to the elimination of high energy component (eye-blink artifact) in the corrected EEG signal. Whereas it is equal to 1 in the $\beta$ band (12–30 Hz), this is due to the fact that energy of eye-blink artifact in this band is very small.

#### Mean absolute error (MAE)

Consider $p_{\mathbf {x}}(f)$ and $p_{\hat{\mathbf {s}}}(f)$ are the power spectrums of the contaminated and the corrected EEG signals, respectively. Then the MAE between the spectrums of both the contaminated and the corrected EEG signals is defined as

11 $$\begin{aligned} MAE=\frac{\sum _{f=l}^{j}|p_{\mathbf {x}}(f)-p_{\hat{\mathbf {s}}}(f)|}{l-j} \end{aligned}$$

where *j* and *l* represent the indices of the start and end frequencies of a specific band. The MAE is computed for the following three bands $1{-}8$, $8{-}12$ and $12{-}30\; \text{Hz}$ to understand affect of artifact removal methods on corrected EEG signals. When there is no loss of EEG components by an artifact removal technique, the MAE value in a band is expected close to zero (in $\beta$ band), which means represents better performance of the artifact removal technique.

### EEG data and pre-processing

To assess the performance of proposed and the existing methods, in this work, we have considered event related potential (ERP) BCI data (ERP-BCI) for both synthetic and real EEG data analysis. The EEG data is obtained from a publicly available database^55,56^ and there is no direct involvement of humans in this research study. The ERP-BCI EEG data^55,56^ is collected from 12 subjects and each subject is asked to spell 20 characters (which results 20 trails) using traditional matrix speller. The EEG signals (64 channels) are recorded using BioSemi Active Two EEG system with sampling frequency 2048 Hz. For this study, we have considered pre-frontal EEG channel $Fp_{1}$. The EEG signals were down sampled to 256 Hz and a band-pass filter with cut-off frequencies $1{-}30 \, \text{Hz}$ is employed to remove dc and high frequency components from the data. More details about the EEG data used in this study are available in^55,56^.
